# Enhanced antimicrobial activity, mechanical and dielectric properties of styrene butadiene rubber vulcanizates designed for protection products

**DOI:** 10.1038/s41598-026-52953-2

**Published:** 2026-06-13

**Authors:** Doaa E EL-Nashar, Fahima Helaly, Aman Khalaf, Azza A. Ward, Nehad N. Rozik, Abbas Yehia, Mohamed M. Eissa, Sahar A. M. Hussein, Abdelmohsen M. Soliman

**Affiliations:** 1 Polymers and Pigments Department, National Research Centre, 33 Elbehouth St., Dokki, Cairo, 12622 Giza Egypt; 2 2Microwave Physics and Dielectric Department, National Research Centre, Giza, Egypt; 3 3Phytochemistry and Plant Systematics Department, National Research Centre, Cairo, 12622 Egypt; 4 Chemistry Department, National Research Centre, Cairo, 12622 Egypt

**Keywords:** Antimicrobial Agents, Styrene Butadiene Rubber (SBR), Chitosan, Ginger, Berberine, Dielectric Properties, Conductivity, Biotechnology, Chemistry, Drug discovery, Materials science, Microbiology

## Abstract

This study focuses on fabricating and evaluating novel, ecofriendly, green, flexible, antimicrobial Styrene Butadiene Rubber vulcanizates for producing safety products. Styrene butadiene rubber (SBR) was compounded with ginger extract, a plant rich in bioactive phenolic compounds, and the conventional drugs, berberine and chitosan, in the ordinary mixer of rubber. Ginger (*Zingiber officinale*) extract was analyzed using HPLC. The rheological properties of the SBR mixes were analyzed to determine the optimal curing time. The compounded rubber mixes were then vulcanized at 152 °C. The prepared vulcanizates were evaluated using Fourier-transform infrared spectroscopy (FTIR), specific surface area measurements, mechanical, swelling, antimicrobial properties, and cytotoxicity analysis. The results showed that the physicochemical and mechanical properties of all vulcanizates remained robust, i.e., no significant degradation, even after exposure to thermal oxidative aging at 90 °C for seven days. Therefore, the change of tensile strength percentage after aging was 25, 5.56, 13, and 10% for free SBR, SBR/30 phr chitosan, SBR/20 phr ginger, and SBR/7 phr berberine, respectively. The cytotoxicity tests confirmed the safety of the investigated vulcanizates due to the negative results against the normal human fibroblast cell line (BJ1). Furthermore, the antimicrobial activity results demonstrated that the release of the various antimicrobial agents successfully inhibited the growth of different bacteria and fungi on the SBR rubber surface. The dielectric and electric findings highlighted the ability of the presence of bioactive agents to modify dielectric relaxation and conductivity in the investigated SBR vulcanizates and confirmed the utility of SBR composites contained 30 phr chitosan, 20 phr ginger, and 7 phr berberine as a good viable choice for antistatic and flexible electronic applications.

## Introduction

The most popular synthetic rubber is styrene-butadiene rubber (SBR), which is made by copolymerizing styrene (25%) and butadiene (75%), using a free radical initiatormechanism^1^. Rubber, without additives, does not show adequate dimensional stability. To provide flexural modulus for SBR rubber vulcanizates, certain additive ingredients, such as oils and fillers, etc., can be added to the rubber composition in addition to the antimicrobial agents to obtain antimicrobial SBR products^2–4^. The rheological, mechanical, physicochemical, and antibacterial qualities are crucial for assessing SBR rubber goods^5,6^. Microbial growth is a serious issue that lowers the quality, safety, and properties of rubber. The antibacterial qualities of SBR rubber goods can be effectively enhanced by incorporating an antimicrobial chemical agent into the ingredients of rubber. The mechanical and antibacterial properties of rubber are thus improved by the crosslinking process to hold the reactive antimicrobial compounds to prevent the growth of microbes while being stored^7–11^.

In pharmaceutical formulations, chitosan (CHI) is a common biocompatible and biodegradable polymer. This polymer’s positive surface charge in an acidic solution is utilized to create tailored nanoparticles^12^. Research has demonstrated that the bacterial membrane’s order is upset and plasma leaks out when chitosan binds to lipopolysaccharides on the bacterial surface^13^.

The chitosan is produced during the deacetylation of chitin^14^. In order to improve the quality of health care for both individuals and populations, it is necessary to identify the structures and processes that influence public outcomes, maintain the structures and processes that support desired outcomes, and change or eliminate those that impede desired goals^15,16^.

Plant extracts have garnered special attention as a natural source of antibiotics. When it comes to its phenolic components, ginger (Zingiber officinale) has demonstrated notable antibacterial properties. It belongs to the Zingiberaceae family, which is the largest and is one of the 10 largest monocotyledonous families used for centuries in Asian medicine. The bioactive compounds that exist in ginger have been identified as phenolic and terpene compounds. The major polyphenol compounds are 6–10. The hydrogenation of gingerols transformed them into corresponding shogaols, which can be transformed by hydration to paradols^17^. The presence of gingerol, a phenolic compound, gives ginger root its spicy flavor. Extracts from ginger exhibit antibacterial action against Escherichia coli and Staphylococcus aureus.

Curcuma, zingiber, chitosan, are examples of natural materials; berberine and thiabendazole (TBZ) are intriguing pesticides with fungicidal properties. Therefore, it is suitable for usage as traditional medicinal medication^18^. Children’s toys, in particular, can host germs, such as E. coli, on dolls and baby dummies or various types of molds growing within rubber ducks. A major factor in product contamination is frequent contact with dirty hands and cleaning supplies. Parents who have little control over the toys their kids play with, particularly in school and the nursery. The current experiment is to manufacture nanocurcumin, incorporate it into rubber compositions using the standard rubber mixer, and investigate the compounded rubber’s rheologically. The recent antibacterial agents used range from approximately 0.001 to 20 weight% of the total weight of rubber, and their specific ingredients^19,20^.

Evaluation of the antimicrobial activity of rubber vulcanizates was done using standard test methods that were applied for plastic (non-porous) and textile (porous) materials, as in ISO 22,196^21^ and the AATCC TM 100^22^. The principles of both standard test methods are actually similar. An inoculated bacterial solution is washed out with sterile neutralizing solution to collect the remaining bacteria for cell culture in a serial dilution manner^23,24^.

However, the limited electrical conductivity and constrained dielectric responses of SBR render it inappropriate for advanced functional applications such as sensors, actuators, and energy storage devices^25^. To reduce these limitations, researchers have explored the incorporation of conductive and polar materials into rubber matrices to alter their dielectric characteristics^26^. Adding antimicrobial agents to SBR can create a material product with both hygiene and electrical insulation properties, expanding its applications into fields where both are required.

Natural additives such as ginger, chitosan, and berberine possess a distinctive combination of ionic characteristics, dipolar groups, and interfacial activity. Ginger contains phenolic compounds and essential oils that may aid in dipolar relaxation and interfacial polarization. Chitosan, a cationic polysaccharide derived from chitin, exhibits proton-conducting characteristics and facilitates charge transport through its amino and hydroxyl groups. Berberine, an isoquinoline alkaloid, possesses conjugated aromatic structures and ionic sites that enhance dielectric responsiveness via space charge accumulation and Maxwell–Wagner–Sillars (MWS) effects^27,28^.

The present work aims to prepare novel antimicrobial SBR vulcanizates containing the investigated active agents like chitosan, berberine, and plant extracts of ginger as primary applied natural antimicrobial agents to produce safety rubber articles and evaluate their mechanical, chemical, dielectric, cytotoxicity, and antimicrobial properties.

## Materials and methods

### Materials

Styrene butadiene rubber (SBR-1502; styrene content 23.5%) from Colon Co., Korea. N-cyclohexyl-2-benzothiazole sulphenamide (CBS), a light gray powder with a melting point of 95–100 °C and a specific gravity of 1.27–1.31 at 25 °C±1) provided by Sigma Aldrich. Stearic acid and Zinc oxide as activators, with specific gravities of 5.55–5.61 and 0.90–0.97 at 15 °C, respectively, were provided by Sigma Aldrich. The elemental sulfur as a vulcanizing agent was applied as a fine, pale-yellow powder with a specific gravity of 2.04–2.06 at ambient temperature, purchased from Sigma Aldrich. Trimethyl-1, 2-dihydroquinoline (TMQ) polymerization, which was employed as an antioxidant, was provided by Sigma Aldrich. Naphthenic oil, with specific gravity 0.94–0.96 at 15 °C, viscosity 80–90 poise at 100 °C, and deep green viscous oil was supplied by Aldrich Company, Germany. Silica as reinforcing filler with specific gravity 1.95, pH 6.2 + 0.8, and contained 82% precipitated silicon dioxide supplied from Degussa, Germany.

#### Antimicrobial Agents

Pharmaceutical drugs or antibiotics, such as Berberine and Chitosan, are purchased from Sigma Aldrich. Plant antimicrobial natural products. Rhizomes of fresh *Zingiber officinale* were purchased from the local market and recognized by Prof. Dr. Sameh R. Hussein. The voucher specimens were placed at the National Research Center, Cairo, Egypt’s herbarium.

Scheme 1 indicates the chemical formula of berberine C_20_H_18_NO_4_. The particle size range from 0.02 –3500 μm. It might help strengthen the heartbeat, which could benefit people with certain heart conditions. Berberine is *an organic heteropentacyclic compound*, an alkaloid antibiotic, a botanical anti-fungal agent, and a berberine alkaloid. *9*,*10-Dimethoxy-5*,*6-dihydro-2 H-7λ*^*5*^*-*^1,3^*dioxolo[4*,*5-g] isoquinolino[3*,*2-a] isoquinolin-7-ylium*.

**Sch. 1:**
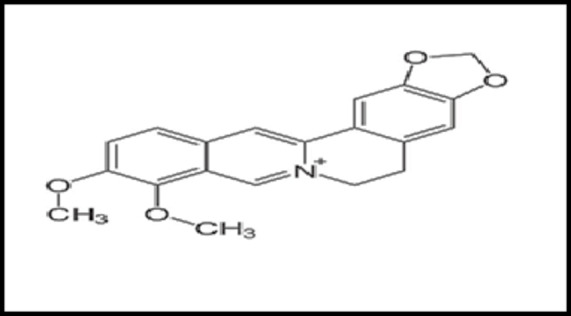
Chemical structure of berberine

Scheme 2 indicates the chemical formula of chitosan with C_56_H_103_N_9_O_39_. Particle size range from 0.01 to 3500 μm. Chitosan is obtained after deacetylation of chitin. The molecules of chitosan consist of an acetylated unit known as N-acetyl-2-amino-2-deoxy-d-glucopyranose and a deacetylated unit known as 2-amino 2-deoxyd-glucopyranose, where the repeating units are linked by β-(1 4)-glycosidic bonds.

**Sch. 2:**
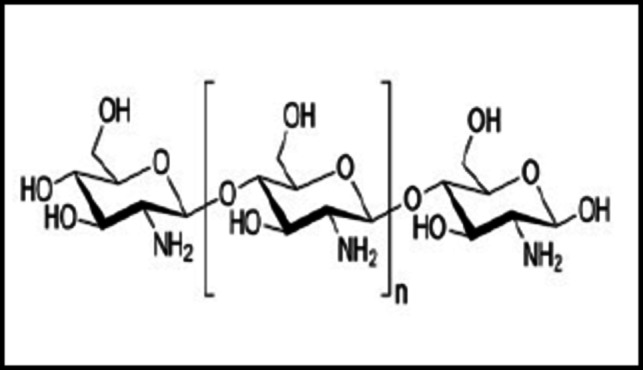
Chemical structure of chitosan

Scheme 3 indicates the chemical formula of Ginger C_17_H_26_O_4_. It is a complex mixture, and its chemical formula varies depending on the specific components present, but the primary active ingredient, 6-gingerol, has the chemical formula C_17_H_26_O_4_. The structure of ginger oil is dominated by sesquiterpenoids, like zingiberene, and monoterpenoids, like citral.

**Sch. 3:**
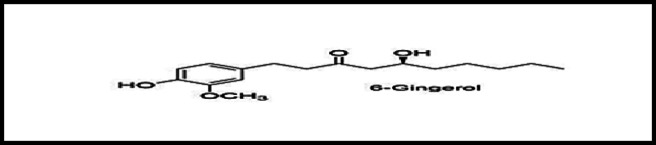
Chemical structure of 6-Gingerol

### Experimental methods

#### Preparation of Plant Extract

Fresh rhizomes were cleaned, cut into thin slices, dried at room temperature, then washed with water and dried, ground into powder.

#### Extraction methods

The collected plant, weighing 250 g, was soaked in 1.5 L of 70% methanol. The aqueous methanol extract was obtained by filtering and vacuum-drying the extracted material, which was collected and dried by rotary evaporation and weighed, resulting in an amorphous dark brown powder for further analysis.

#### Determination of total flavonoid content

Plant extract (0.1 mg/mL) was diluted with 4 mL of water. Each volumetric flask was filled with a 5% NaNO_2_ solution of 0.3 mL for 5 min, followed by 10% AlCl_3_ (w/w) for six minutes, then 2 mL of NaOH (1.0 M) was added to the volumetric flask and 2.4 mL of distilled water, then thoroughly mixed in the reaction flask. At ƛ430 nm, the absorbance was measured. The results were calculated in mg quercetin as standard.

#### Quantitative analysis using HPLC method

The Technologies Company (Shimadzu, Japan) was used to analyze *Zingiber officinale* extract. The HPLC column is a Zorbax RP_18_ column with a photodiode detector, and DAD was used^29^. Ginger extract was dissolved in methanol HPLC grade, the gradient elution was used with the eluent A: water with 0.1% formic acid addition, the eluent B: acetonitrile (flow rate is 0.5 mL/min with injection volume of 5 µL) solvent A to B from 0 to 100% gradient for 40 min. The DAD detector was 160–380 nm. The Quantitative determinations for active phenolic compounds, which were presented in the methanol extract in major amounts. From the comparison between the retention time of the slandered sample and major compounds, identify: 6-gingerol, 8-gingerdione, 8-shogaol, and 10-gingerdione.

#### Rubber formulas and compounds

Table 1 lists the constituent parts of SBR rubber formulations and its ingredients, including the antimicrobial agents under investigation. The most effective mixing order for developing compounded SBR rubber as follows: Masticate the rubber to combine with the other ingredient materials; it was initially passed twice without bands at a roll aperture of roughly 0.2 mm. After that, inserted into a laboratory two-roll mill with an outer diameter of 470 mm and a working distance of 300 mm through a mill aperture of around 1.5 mm. The friction gear ratio was set at 1.4:1 for 1.5 min while the slow roll was operated at 24 r min^− 1^. It takes roughly 3.5 min to add antimicrobial chemicals in situ using the masticated SBR produced surrounding the roll mill. The mixing period was followed by the addition of the remaining properly weighed additives, which included sulfur vulcanizing agent, processing oil, accelerators, activators, and antioxidants, in a consistent order. As per ASTM D-3182, the rolls were cut into three or four pieces on each side. Before molding, the resulting rubber mixes were sheeted to create sheets that were at least six hours old and 1–2 mm thick. After mixing, the sample weights were ultimately verified. According to Mackey Bowley (C1136199), the compounded SBR mixes were compressed molded (vulcanized) in an electrically heated hydraulic press at 152 ± 1 °C under 4 MPa pressure. According to ASTM D-1928, the rheometer was used to determine the optimal curing time, at 152 ± 1 °C.

**Table 1:** Formulations of SBR containing various types and amounts of antimicrobial agents.

Ingredients	Role	Content (phr)^a^										
		S0	Ch10	Ch20	Ch30	Ch50	Gin10	Gin20	Gin30	Beb5	Beb7	Beb10
SBR Stearic acid Zinc oxide TMQ^b^ Sulfur CBS^c^ Silica Naphthenic oil	Rubber Activators Activators Antioxidant Curing agent Accelerators filler Plastisizer	100 2 5 1.5 1.5 1.2 30 2	100 2 5 1.5 1.5 1.2 30 2	100 2 5 1.5 1.5 1.2 30 2	100 2 5 1.5 1.5 1.2 30 2	100 2 5 1.5 1.5 1.2 30 2	100 2 5 1.5 1.5 1.2 30 2	100 2 5 1.5 1.5 1.2 30 2	100 2 5 1.5 1.5 1.2 30 2	100 2 5 1.5 1.5 1.2 30 2	100 2 5 1.5 1.5 1.2 30 2	100 2 5 1.5 1.5 1.2 30 2
**Chitosan (Ch)**	**Antimicrobial agent**	**-----**	**10**	**20**	**30**	**50**	**-----**	**-----**	**-----**	**-----**	**-----**	**-----**
**Ginger (Gin)**	**Antimicrobial agent**	**-----**	**-----**	**-----**	**-----**	**-----**	**10**	**20**	**30**	**-----**	**-----**	**-----**
**Berberine (Beb)**	**Antimicrobial agent**	**-----**	**-----**	**-----**	**-----**	**-----**	**-----**	**-----**	**-----**	**5**	**7**	**10**

^a^ Part per hundred parts of rubber. ^b^ polymerized 2,2,4-trimethyl-1,2-dihydroquinoline. ^c^ N-cyclohexyl-2-benzothiazole sulfonamide.

### Characterization and measurements

#### Fourier transforms infrared spectroscopy (FTIR) technique

Rubber vulcanizates infrared spectroscopy with antimicrobial agents was performed using a Jasco FTIR-430 series infrared spectroscopy fitted with KBr discs.

#### Rheological characteristics

The Moving Die Rheometer (MDR 1), USA, is a TA device. Rheometric characteristics were assessed using the standard method of ASTM D2084 at 152 ± 1 °C for determined scorch times (ts_2_, min), optimum cure time (tc_90_, min), minimum torque (M_L_), maximum torque (M_H_), and cure rate indices (CRI, min^− 1^).

#### Scanning electron microscope (SEM)

Scanning electron microscope (SEM) is a useful instrument for examining sample morphology surfaces and the dispersion of investigated active antimicrobial agents. Utilizing a Quanta FEG-250 to take SEM pictures of the samples, the surface morphology of the finished SBR compounds was investigated. Over the period of six minutes, a very tiny layer of gold, five to ten nanometres thick was applied to the samples, to provide a representative sample of the specimen’s general shape. The centre cross-section was chosen.

#### Mechanical characteristics

Styrene butadiene rubber (SBR) sheets cut with an ASTM Wallace die cutter have physical-mechanical characteristics. Using the vulcanizates from the molded sheets, dumbbell-shaped specimens were created. The results are based on the average of five different dumbbell-shaped specimens with an estimated thickness of 1.5 mm and a working portion of 15 mm, and a consistent 4 mm width. A common thickness gauge was used to measure each specimen thickness. Rubber nanocomposite vulcanizates were tested for the mechanical properties using a Zwick/Roell Z010 tensile tester machine with a load cell (Type: X force P and Nominal force: 10 KN) was used to measure the tensile strength (MPa), elongation at break (%), and modulus at 50, 100, and 200% elongation, accordance with ASTM D412. Five replicates of the mechanical data were measured for the average.

#### Equilibrium swelling (Q)

The equilibrium swelling procedure was followed in accordance with ASTM D471. The specimens were immersed in toluene for 24 h at room temperature (25 ± 1 °C) in order to induce the swelling. The apparent percentage change in mass was calculated; the percentage of swelling was calculated according to Eq. (1).

1 $$Q\% {\text{ }}={\text{ }}[\left( {w - w`} \right)]{\text{ }}x{\text{ }}100$$

w is the sample initial weight, w` is the sample ultimate weight after swelling.

#### Thermal oxidative aging

Accelerated thermal aging was done in an electric oven at 90 ^**º**^C for 7 days.

#### Broadband Dielectric Spectroscopy

Schlumberger Impedance / Gain-Phase Analyzer 1260 in the frequency range 0.1 Hz to 1 MHz was used to obtain the permittivity ε′, dielectric loss ε″, and alternating resistance R_ac_ at room temperature (30 ± 1 °C). Three samples are utilized for each measurement, and the average is then calculated. The error in ε′ and tan δ amounts to ± 1% and ± 3%, respectively. The temperature of the samples was controlled by a temperature regulator with a Pt 100 sensor. The error in temperature measurements amounts to ± 0.5 °C.

### Investigation of the quality and efficiency of the prepared antimicrobial rubber products

#### Bioassay Tests for the antimicrobial rubber products

The activity of antimicrobial rubber formulations on normal human skin cell lines and different pathogenic microbes will be investigated to evaluate the safety and efficacy of the investigated formulations for the manufacturing of antimicrobial personal protection products^30^.

#### Cytotoxicity activity assay

The newly prepared rubber vulcanizates containing antiviral and antimicrobial agents will be tested towards the normal human skin cell line: BJ1 Fibroblast using MTT assay^31^.

#### Antimicrobial Activity

##### Determination of Antimicrobial Finished Products

The antimicrobial activity of the finished rubber vulcanizates was evaluated according to the International Standard ISO 20,743^32–34^. This method quantifies the ability of antimicrobial-treated products to inhibit or kill test microorganisms over an incubation period of 18 to 24 h.

ISO 20,743 specifies three quantitative test procedures:

1. Absorption method.
2. Printing method.
3. Transfer method.

In the present study, the Transfer method was employed. Briefly, the surface of an agar plate was uniformly inoculated with a standardized suspension of the test bacterium. A test specimen (SBR rubber vulcanizate, with or without antimicrobial agents) was then placed onto the inoculated agar surface and pressed down under a defined weight for 60 s to ensure contact. Subsequently, the specimen was incubated under humid conditions for 18–24 h. Viable bacterial concentrations on the specimen were determined before and after incubation to calculate the antimicrobial activity.

The Printing method, also described in ISO 20,743, involves printing membrane-filtered bacteria onto the test specimen prior to incubation under humid conditions for 1 to 4 h. Although available, this method was not used in the current study.

The adoption of the Transfer method was motivated by the need for an ISO-compliant procedure applicable to a wide range of final products, including rubber and textiles. The method is based on the Japanese Industrial Standard (JIS) approach, ensuring consistency with established protocols.

## Results and discussion

The highest total phenolic content was obtained from chloroform: methanol (1:1, v/v) extract (59.23 ± 0.43 mg gallic acid/g) of rhizome. The Flavonoids were detected in rhizome chloroform: methanol extract (44.26 ± 0.20 mg quercetin/g). Rhizome extracts exhibited good antioxidant activity.

### HPLC analysis

Separation of compounds was conducted using HPLC. By comparing retention times (Rt) with standards and UV absorbance ratios after injection of samples and standards, it was possible to identify the phenolic compounds present in the extract. By using the standard calibration curves, the amounts of gallic acid, caffeic acid, naringin, rutin, and quercetin were determined as in Scheme 4.

**Sch. 4:**
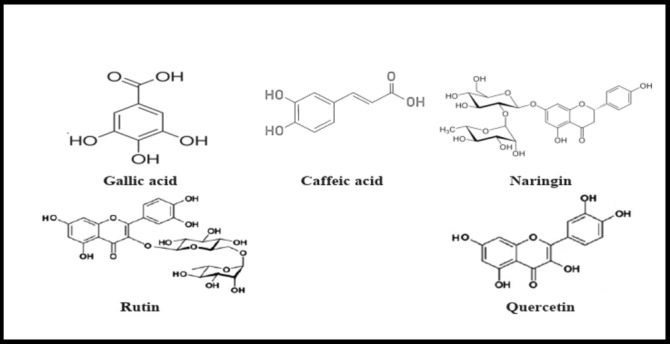
Chemical structures of identified bioactive compounds: gallic acid, caffeic acid, naringin, rutin, and quercetin

Peak identification based on the standard injection: Gallic acid (R_t_: 3.345 min); caffeic acid (R_t_: 6.641 min); naringin (R_t_: 8.533 min); rutin (R_t_: 9.356 min); and quercetin (R_t_: 12.739 min), Fig. 6a.

The Quantitative determinations for active phenolic compounds, which were presented in the methanol extract in major amounts, are shown in Scheme 5.

From comparison between retention time of slandered sample and major compound, identify: 6- gingerol at R_t_:16.62 min; 8-gingerol at R_t_: 20.84 min; 8-gingerdione at R_t_: 21.63 min; 8-shogaol at R_t_: 22.91 min; 6-dehydrogingerdione at R_t_: 25.02 min; 10-gingerdione at R_t_: 25.80 min and 8-dehydrogingerdione at R_t_: 30.12 min, the values of the content of active substances in the ginger extract calculated based on the HPLC analysis are presented, Fig. 6b.

**Sch. 5:**
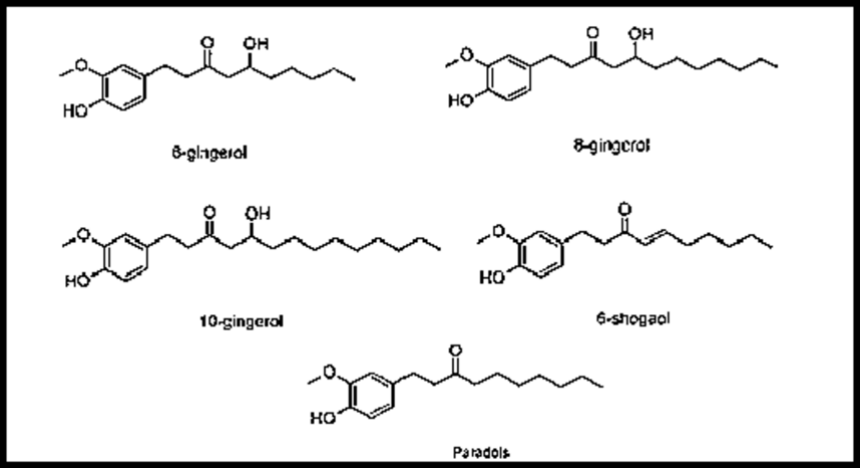
structure of identified phenolic compounds: 6-gingerol; 8-gingerol; 8-gingerdione; 8-shogaol; 6-dehydrogingerdione; 10-gingerdione and 8-dehydrogingerdione.

**Fig. 1:**
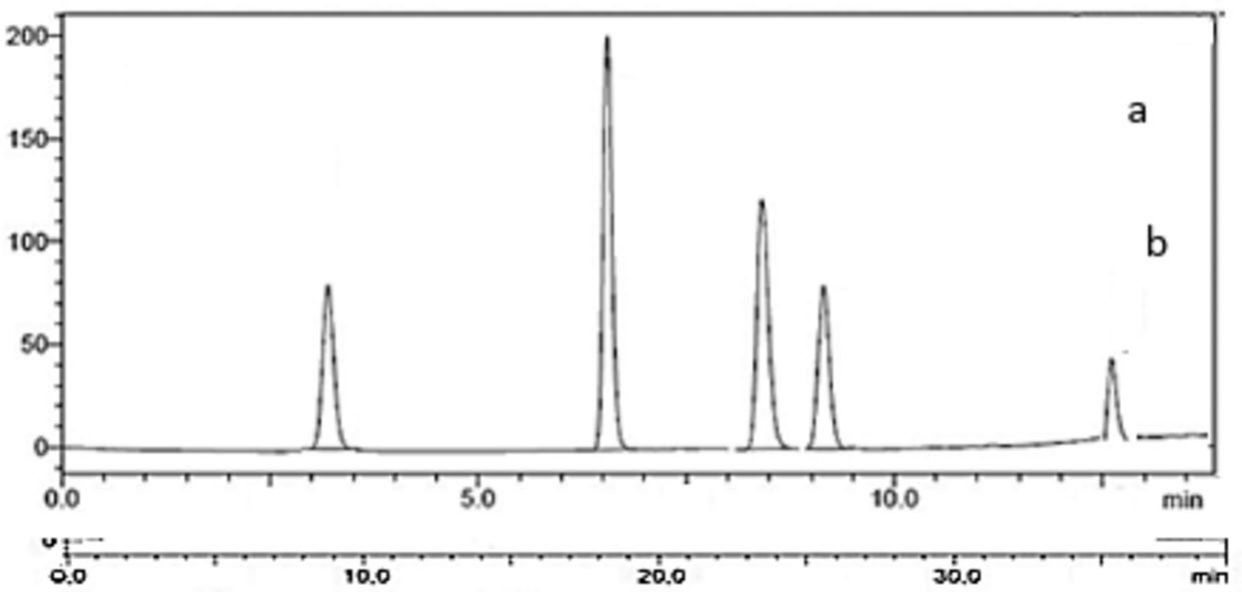
HPLC chromatogram of: **a** - ginger extract with identified bioactive compounds, **b**- ginger extract with identified active compounds.

### Rheological properties

The processability of composites was determined by evaluating the rheological characteristics such as minimum torque M_L_, maximum torque M_H_, scorch time ts_2_, and optimum cure time tc_90_. Determination of the curing time is a prerequisite for molding the rubber compounds. The curing or vulcanization time was determined for each prepared formula.

The rheometric characteristics of the examined SBR vulcanizates are illustrated in Table 2.

A moving die rheometer was used to measure the compounded SBR rheological characteristics at 152 °C. The results are listed in Table 2. The rheological characteristics of SBR were determined to be good. The gathered rheological data used to determine the curing time of the prepared compounded rubber. In the case of SBR-contained chitosan, plant extracts Ginger as well as pharmaceutical medicine Berberine as conventional antimicrobial agents the minimum and maximum torque increased according to their concentrations; the M_L_ and M_H_ for Ginger increased up to 10 phr then decreased, nevertheless, the addition of investigated antimicrobial agents enhanced the minimum torque of SBR, M_L_ and M_H_ values are higher than those of SBR combined with chitosan, ginger and pharmaceutical medicine Berberine, suggesting a minor decline in the cure state; however, 50 phr chitosan incorporated with SBR has higher M_L_ and M_H_ values^22^. The rate of cure for SBR combined with chitosan and Berberine increased due to their concentrations, but for ginger increased up to 10phr. The combination of SBR (Styrene Butadiene Rubber) with chitosan, ginger, and Berberine can lead to an increased rate of cure and improve their rheological properties. Additionally, the overall studies showed a reduction in optimal cure time (tc_90_), resulting in good, acceptable rheological properties. It indicates improved curing efficacy and processability, which produced high-quality products^35^.

On the other hand, scorch time ts_2_ of rubber mixes decreased with increasing chitosan content. The decrease in scorch time ts_2_ as the chitosan content increased may be due to the greater thermal history of these compounds during mixing as a result of their higher viscosities. It is known that the shear heating during mixing increases when chitosan loading is increased due to the increase in viscosity. But ts_2_ increased as the ginger content increased up to 10phr, then decreased. Also, ts_2_ increased as the Berberine content increased, although adding more ginger will initially lengthen the scorch time. The time takes for SBR rubber to begin vulcanizing makes it less pliable and shortens the scorch time, triggering vulcanization sooner. This is probably because of the intricate relationships between ginger and other ingredients in the rubber compound during vulcanization^22,36^.

The presence of fillers that aid in the activation of curing reactions causes the Cure Rate Index (CRI) to rise^19^.

**Table 2:** Rheometric properties of SBR loaded with Chitosan, Ginger, and Berberine at different doses as antimicrobial agents.

Sample keys	S0	Ch 10	Ch 20	Ch 30	Ch 50	Gin 10	Gin 20	Gin 30	Ber 5	Ber 7	Ber 10	Mean	SD
Rheological properties													
(M_L_), dN_m_	2.67	3.23	3.50	3.58	4.96	3.02	2.84	2.06	3.33	3.50	3.63	**3.30**	**0.73**
(M_H_), dNm	21.43	29.56	32.56	42.38	43.26	21.95	17.22	19.64	23.55	27.48	29.43	**28.04**	**8.67**
(t_S2_), min	0.75	0.56	0.50	0.46	0.45	1.35	1.10	0.93	1.37	8.05	4.01	**1.78**	**2.31**
(t_C90_), min	34.12	29.37	28.98	27.83	26.16	22.24	19.61	16.86	21.15	12.49	7.02	**22.35**	**8.05**
(CRI)	3.00	3.47	3.51	3.65	3.89	4.79	5.40	6.28	5.06	22.52	33.22	**8.62**	**9.86**

Minimum torque (M_L_), Maximum torque (M_H_), Scorch time (ts_2_), Optimium Cure Time (t_C90_), Cure rate index (CRI).

### Fourier transforms infrared spectroscopy (FTIR)

Figure 7 presents the IR spectra of SBR composites containing chitosan, ginger, and berberine. The characteristic bands of SBR are evident, confirming the presence of styrene and butadiene units, with C = C stretching at ~ 1600 cm⁻¹ (aromatic ring and butadiene double bonds) and = C–H out-of-plane bending in the 1000–900 cm⁻¹ region (vinyl groups and aromatic C–H of styrene)^37^. Incorporation of chitosan is confirmed by new absorption bands related to –OH, –NH₂, and polysaccharide linkages, together with the appearance of amide (~ 1650 and 1590 cm⁻¹) bands. The broadening of the O–H/N–H stretching region (3200–3400 cm⁻¹) further indicates hydrogen bonding between chitosan and SBR^38^. These changes indicate hydrogen bonding between chitosan’s amino/hydroxyl groups and the SBR chains, enhancing compatibility. The presence of ginger phytochemicals is reflected in the stretching of phenolic –OH groups at 3300–3200 cm⁻¹, along with C = O stretching bands at 1700–1650 cm⁻¹ (ketones, aldehydes) and aromatic C = C vibrations. These peaks confirm the successful incorporation of ginger into the SBR matrix^39^. These features confirm the presence of ginger extract and suggest physical interaction with the SBR matrix rather than chemical bonding. For berberine, characteristic vibrations of aromatic amines (C–N stretching) appear in the 1450–500 cm⁻¹ region, while enhanced C–N and C–O peaks (1250–1020 cm⁻¹) arise from its methoxy and alkaloid structures^40^. The changes in band intensity and minor shifts imply π–π interactions and possible weak hydrogen bonding between berberine molecules and SBR chains. Overall, the observed shifts, broadened bands, and additional absorption features confirm the successful incorporation of the bio-additives into SBR. These spectral changes suggest intermolecular interactions—including hydrogen bonding, π–π stacking, and possible ionic interactions—between SBR and chitosan, ginger, and berberine. The main SBR peaks do not significantly shift, confirming absence of chemical degradation. Additive-specific peaks appear clearly, confirming successful loading. Minor shifts in the –CH and C = C peaks imply interfacial interactions between SBR and the bio-fillers.

**Fig. 2:**
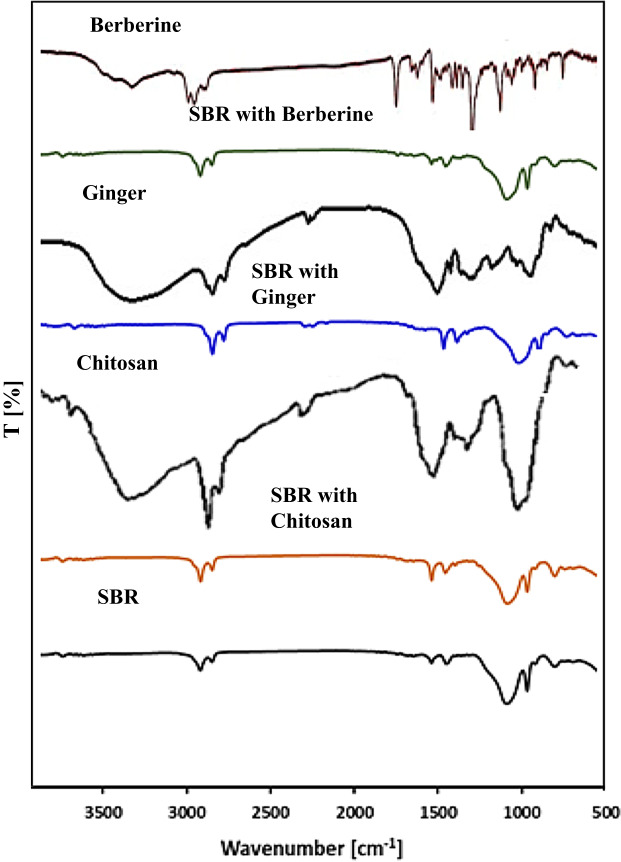
FTIR Spectra of SBR with chitosan, ginger, and berberine.

### The physico-mechanical properties

It is interesting to evaluate **the** physico-mechanical properties of the SBR vulcanizates containing the bioactive materials according to ASTM 412.

Table 3 shows the mechanical properties of the SBR vulcanizates under investigation. According to the obtained results, the SBR vulcanizates that were tested exhibited good mechanical qualities. When chitosan, plant extracts like ginger, and traditional pharmaceutical drugs like berberine were present, it was demonstrated that, the mechanical properties, tensile strength, elongation at break, and modulus at 100, 200, 300, and 500% were enhanced. Tensile strength of SBR vulcanized with chitosan increased as the percentage of chitosan increased, due to the presence of NH2 amino group and OH group^41^, that enhanced mechanical strength and also the interfacial adhesion between SBR and chitosan increased and consequently strength increased, then decreased at 50phr, which may be due to the phase separation between the vulcanized SBR phase and chitosan phase^42^. Also, the presence of ginger in the SBR vulcanizates increases the tensile strength up to a concentration of 20phr, then decreases at 30phr, but still more than SBR vulcanizates without ginger. Therefore, the incorporation of ginger in SBR vulcanizates enhances the tensile strength and all mechanical properties and can be utilized for the antimicrobial rubber articles^43^. Also, the incorporation of berberine into the SBR results in an increase in tensile strength in comparison with the SBR blank. The highest value of tensile strength was obtained at 7phr berberine, and then decreased at 10phr. This can be attributed to the fact that berberine significantly changes the degree of crystallinity as well as forming a unique type of network that reinforces the structure of the obtained materials^35,44^. On the other hand, the elongation at break for SBR vulcanizates containing different concentrations of chitosan and ginger (10,20,30phr) was increased more than the control one (SBR free) or did not change depending on the increasing concentrations after 30phr, so the elasticity of the vulcanizates was good. Elongation at break was significantly increased after the introduction of berberine, this is due to the good dispersion and homogeneity of the Berberine through the SBR matrix, which was confirmed by SEM analysis^45^. According to berberine, concentration increased the elasticity of the vulcanized rubber, up to 7phr, and then decreased. The lowest value of elongation at break of vulcanized rubber containing 10 phr berberine was obtained. This indicates that the elongation at break of the SBR sample with 7phr is greater than that of the SBR without berberine^46^.In addition, the modulus changes for the investigated vulcanizates at various elongations (50–500%) were not significantly different from the control one^47,48^. Consequently, the investigated antimicrobial agents can help to improve the mechanical characteristics; this was confirmed with the previously reported work^36,38^. Since they produce rubber vulcanizates with superior homogeneity and high dispersion^35,49,50^.

**Table 3:** Mechanical properties of SBR vulcanizates loaded with various concentrations of different antimicrobial agents.

Sample keys	TS (MPa)	E (%)	M, 50% (MPa)	M,100% (MPa)	M, 200% (MPa)	M, 300% (MPa)	M, 500% (MPa)
S0	9.64	450	1.49	1.82	2.11	2.37	2.99
Chitosan 10	13.66	555	2.24	2.27	2.55	3.21	3.91
Chitosan 20	15.32	580	2.76	2.98	3.40	3.72	4.25
Chitosan 30	15.44	583	3.51	4.24	5.09	5.63	6.24
Chitosan 50	8.94	430	1.05	1.28	1.58	1.92	2.96
Ginger 10	12.83	553	1.77	1.99	2.28	2.60	3.39
Ginger 20	13.83	570	2.25	2.29	2.67	3.98	3.96
Ginger 30	10.87	480	1.95	2.03	2.15	2.41	3.05
Berberine 5	12.13	550	1. 71	2.15	2.28	2.59	3.32
Berberine 7	14.07	560	2.68	2.83	2.93	3.33	4.22
Berberine 10	11.24	530	1.95	2.11	2.19	2.49	3.11
**Mean**	**12.54**	**531**	**2.12**	**2.36**	**2.66**	**3.11**	**3.76**
**SD**	**2.18**	**53.15**	**0.68**	**0.77**	**0.94**	**1.04**	**0.96**

Tensile Strength (TS), Elongation at break (E), Modulus (M).

### Chemical properties

#### Equilibrium swelling

**Fig. 3:**
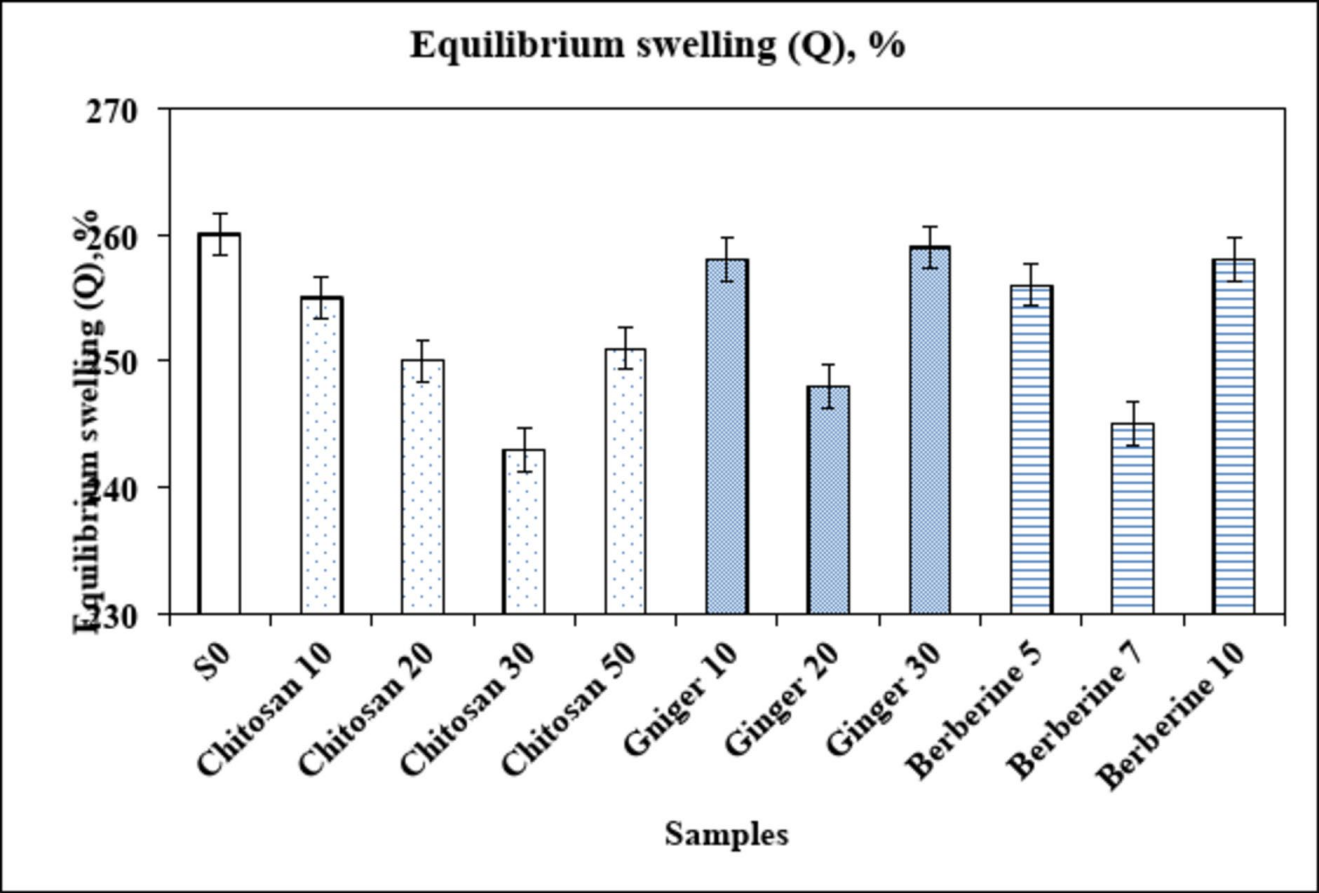
Equilibrium swelling of SBR vulcanizates loaded with different antimicrobial agents at different concentrations.

The equilibrium swelling properties are demonstrated in Fig. 3, which shows the equilibrium swelling of the SBR vulcanizates containing the antimicrobial medications under investigation. The equilibrium swelling of all SBR vulcanizates was demonstrated to be good. The proportion of swelling was reduced at chitosan (10, 20, 30 phr), ginger (10, 20 phr), and berberine (5,7 phr) concentrations. Because chitosan, ginger, and berberine inhibited toluene from penetrating the prepared samples, the proportion of edema was reduced. Thus, the cross-linking increased, the swelling percentage decreased, and the qualities of SBR rubber vulcanizates improved^35,49^.

Therefor the mechanism of vulcanization illustrated as the following diagram Scheme 6, while the investigated active compounds are physically bonded with the vulcanized SBR matrix or holding between cross links^35,49^.

**Sch. 6:**
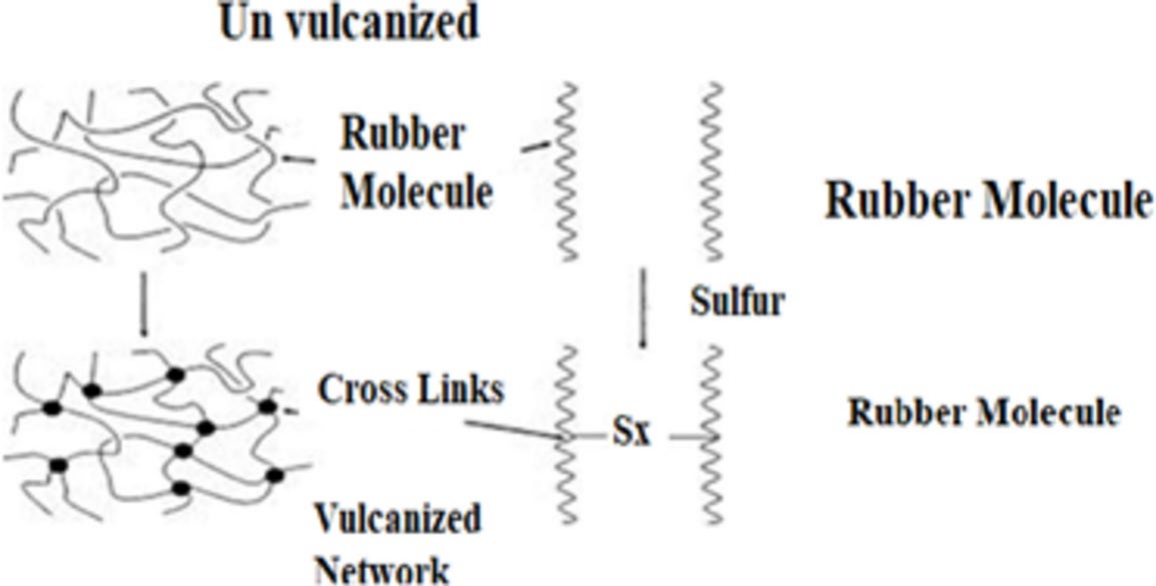
structure of identified phenolic compounds: 6-gingerol; 8-gingerol; 8-gingerdione; 8-shogaol; 6-dehydrogingerdione; 10-gingerdione and 8-dehydrogingerdione.

### Thermal oxidative aging

**Fig. 4:**
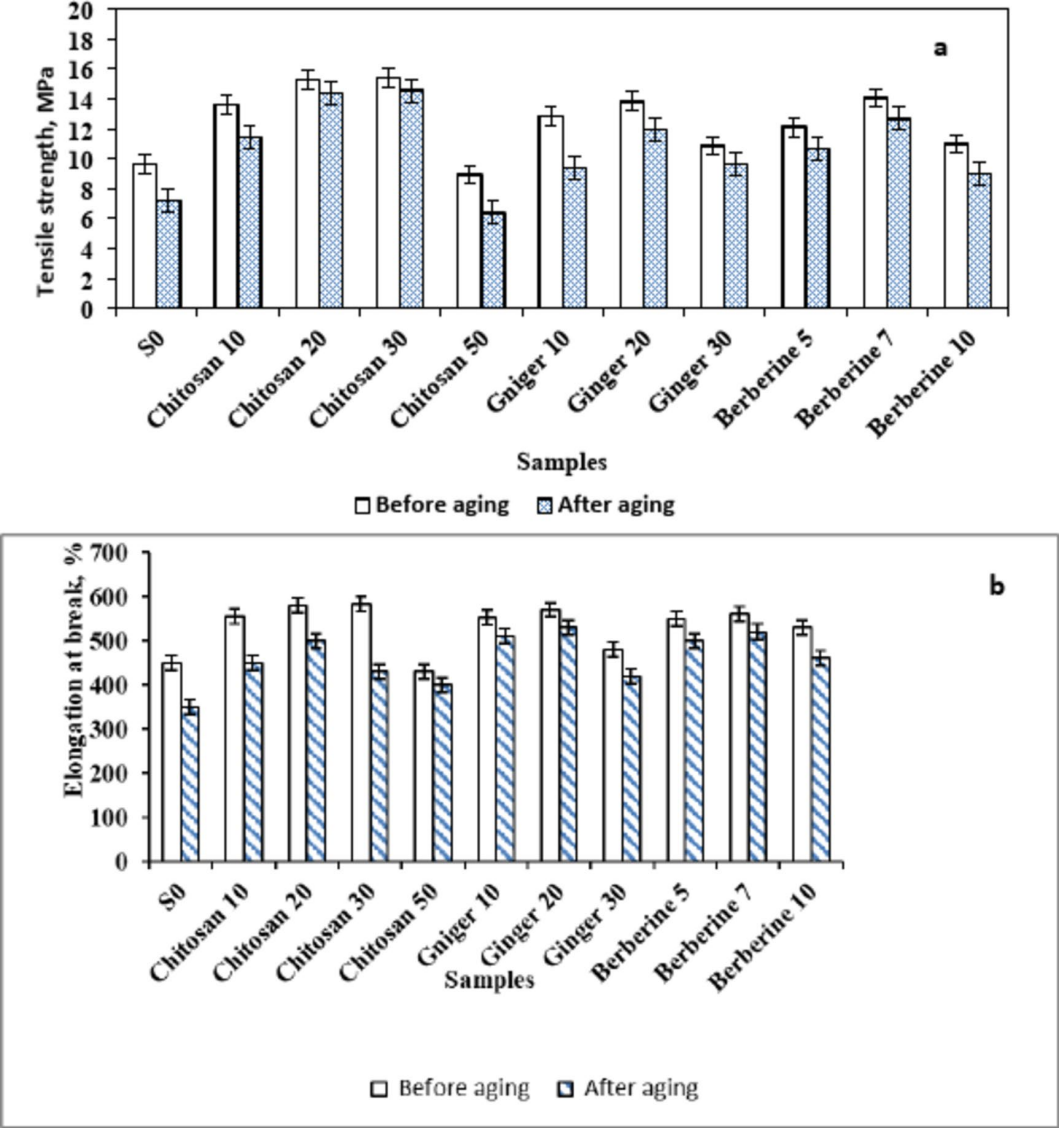
Tensile strength (a) and elongation at break % (b) of the SBR vulcanizates before and after six days of aging at 90 °C with varying concentrations of distinct antimicrobial agents.

For improved rubber product service performance, rubber vulcanizates must be resistant to heat oxidative aging. The prepared vulcanizates, tensile characteristics were assessed following exposure to aging in order to assess the potential of adding plant extracts and traditional pharmaceutical medications as antimicrobial agents to the SBR vulcanizates withstand the effect of the aging process on the prepared SBR composites. All examined SBR vulcanizates containing bioactive ingredients (chitosan, ginger, and berberine) showed a greater resistance to thermal oxidative aging, meaning they had favorable qualities both before and after being aged for six days at 90 °C, as shown in Fig. 4 (a, b). When comparing the properties to the control, there is little variation in the values of elongation at break and tensile strength. These outcomes may be the consequence of the active ingredients’ nature, which inhibits radicals and delays aging to provide long-term protection^50,51^.

### Scanning electron microscopy (SEM)

**Fig. 5:**
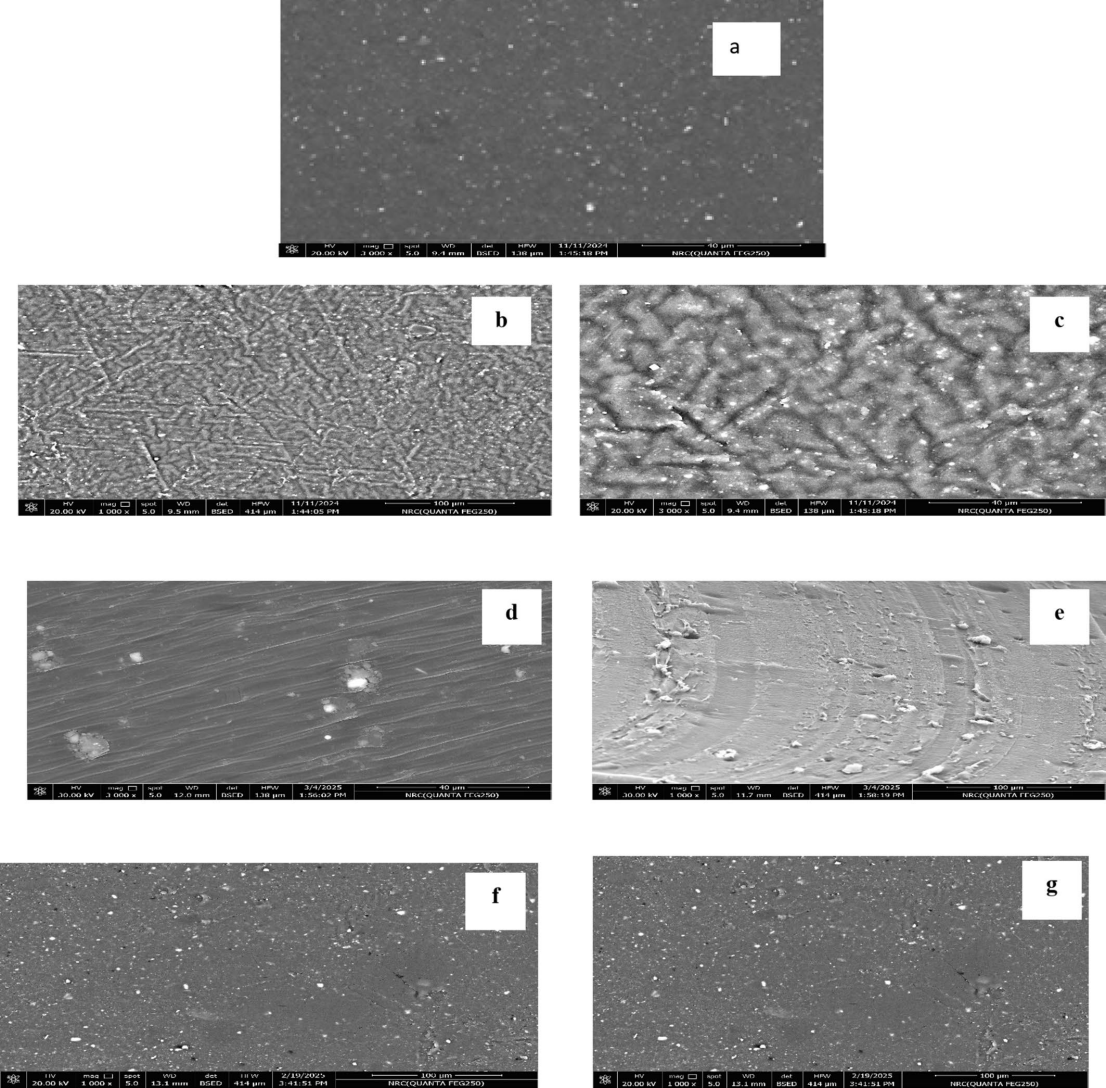
Scanning electron microscope for SBR vulcanizates in the absence and presence of antimicrobial agents: (a) SBR, (b) SBR/30 phr chitosan, (c) SBR/50 phr chitosan, (d) SBR/20 phr ginger, (e) SBR/30 phr ginger, (f) SBR/7 phr berberine, (g) SBR/10 phr berberine.

The SEM images in Fig. 5 (a, b, c, d, e, f, g) show important information about the morphology of SBR vulcanizates, as well as the compatibility and dispersion of antimicrobial agents. The sample surfaces are comparatively smooth and uniform. The SEM image of SBR without antimicrobial agents usually shows a smooth and relatively featureless surface. It might have some minor irregularities or particle agglomerations. A homogeneous distribution of the investigated antimicrobial agents at 30 phr chitosan, 20 phr ginger, and 7 phr berberine concentrations is visible as in Fig. 5 (b, d, f), which depicts good surface homogeneity due to the presence of interfacial bonding. Enhancing interfacial bonding can strengthen the SBR matrix and improve the mechanical properties of the samples. The micrographs depict the good dispersion and distribution of the antimicrobial agents inside the SBR rubber matrix. The antimicrobial agents particles exhibit a discernible aggregation as in Fig. 5 (c, e, g), which illustrates that the SBR containing 50 phr chitosan, 30 phr ginger, and 10 phr berberine had some uniform dispersion of antimicrobial agents and presence of some aggregation due to increasing the concentration. So, the suitable concentration to obtain a homogeneous surface and good dispersion of the active ingredients is less than those concentrations. This clustering may result in weak spots in the SBR vulcanizates, which would impair mechanical performance and load transfer^19,52^. Poor dispersion, however, can lead to stress concentration at some points and reduce the overall performance of the vulcanizates.

### Dielectric properties

**Fig. 6:**
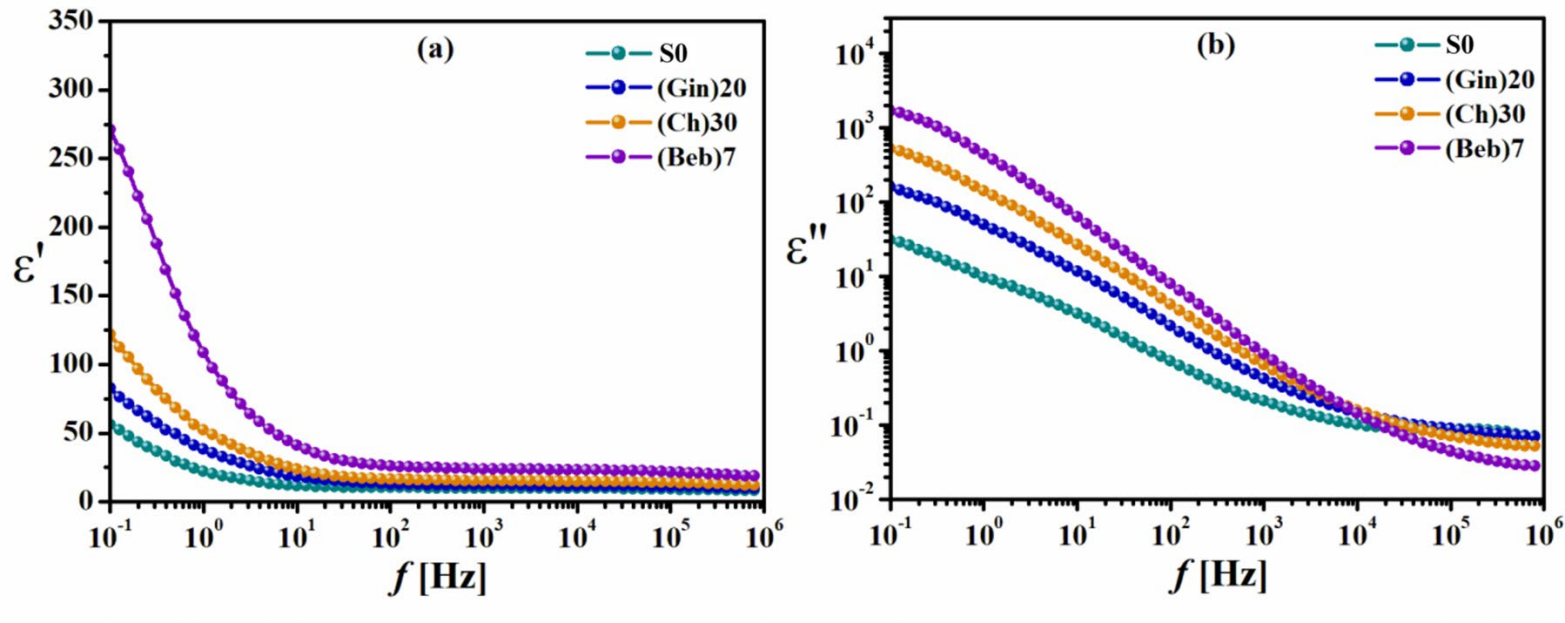
Frequency-dependent dielectric behavior of SBR-based composites; (**a**) Permittivity (ε′), and (**b**) Dielectric loss (ε″) at 30 °C.

Dielectric investigations revealed the optimal concentrations for each antimicrobial agents, selected for their improved mechanical performance. Figure 6a illustrates the frequency-dependent dielectric constant (ε′) of SBR-based vulcanizates, while Fig. 11b presents the corresponding dielectric loss (ε″). All samples exhibit a typical decrease in ε′ and ε″ with increasing frequency, consistent with dipolar relaxation and reduced interfacial polarization at higher frequencies^53^. The SBR vulcanizates loaded with 7phr berberine demonstrates the highest ε′ and ε″ values throughout the frequency spectrum, indicating substantial space charge accumulation and ionic polarization effects due to the conjugated and ionizable nature of berberine^54^. Whereas SBR vulcanizate loaded with 20 phr of ginger exhibit a moderate enhancement in both parameters. The polar phenolic compounds and essential oils in ginger likely facilitate dipolar alignment in the presence of an electric field^55^. In addition, the vulcanizate (Ch) 30 displays intermediate dielectric properties due to the contribution of chitosan’s amino and hydroxyl groups, which facilitate protonic conduction and the formation of interfacial dipoles^56^. The free from bioactive materials; SBR sample (N0) demonstrates the lowest dielectric response, thereby confirming the role of bioactive agents in modifying the electrical properties of the matrix. The findings reveal that berberine markedly enhances both permittivity and dielectric dissipation, positioning the composite (Beb)7 as a suitable candidate for applications requiring outstanding dielectric performance.

**Fig. 7:**
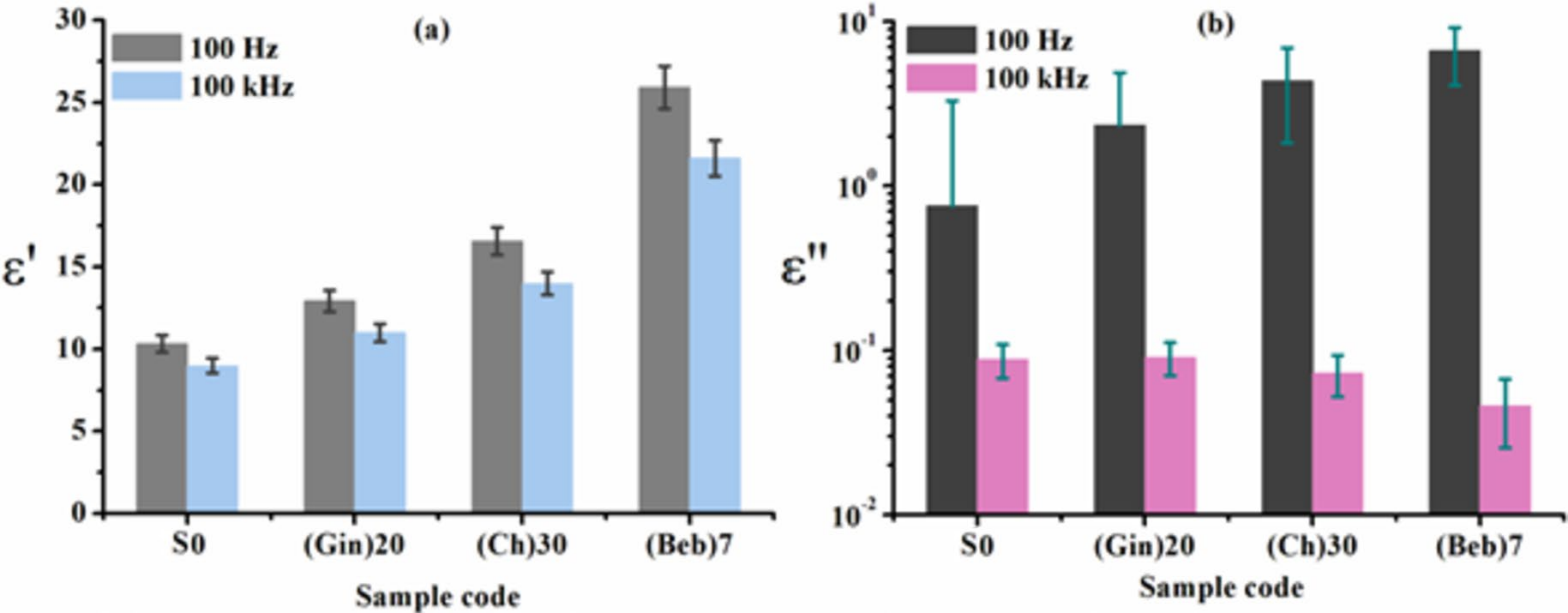
Variation of (**a**) Permittivity (ε′), and (**b**) dielectric loss (ε″) for SBR-based composites at two specified frequencies: 100 Hz and 100 kHz, respectively.

Figure 7 depicts the dielectric properties of SBR-based composites at two specified frequencies: 100 Hz and 100 kHz. Figure 7a illustrates that the values of permittivity ε′ are much higher at 100 Hz compared to 100 kHz across all samples.Dipolar and interfacial polarization are more pronounced at low frequencies^57^. The vulcanizate (Beb)7 exhibits the highest ε′ values, consistent with berberine’s ionic and conjugated structure that facilitates space charge accumulation and Maxwell–Wagner–Sillars (MWS) polarization^54^. The composites (Gin)20, and (Ch)30 have intermediate values,attributable to the polar constituents of ginger and the proton-conducting amino groups in chitosan, respectively^56,58^. The sample N0 (blank sample) has the lowest permittivity ε′, indicating that bioactive fillers enhance dielectric response.

On the other hand, Fig. 7b depicts the dielectric loss ε″ for SBR composites. At 100 Hz, all samples exhibit much higher ε″ values compared to those at 100 kHz. Ionic conduction and dipolar relaxation are the primary mechanisms of energy dissipation at lower frequencies^59^.

**Fig. 8:**
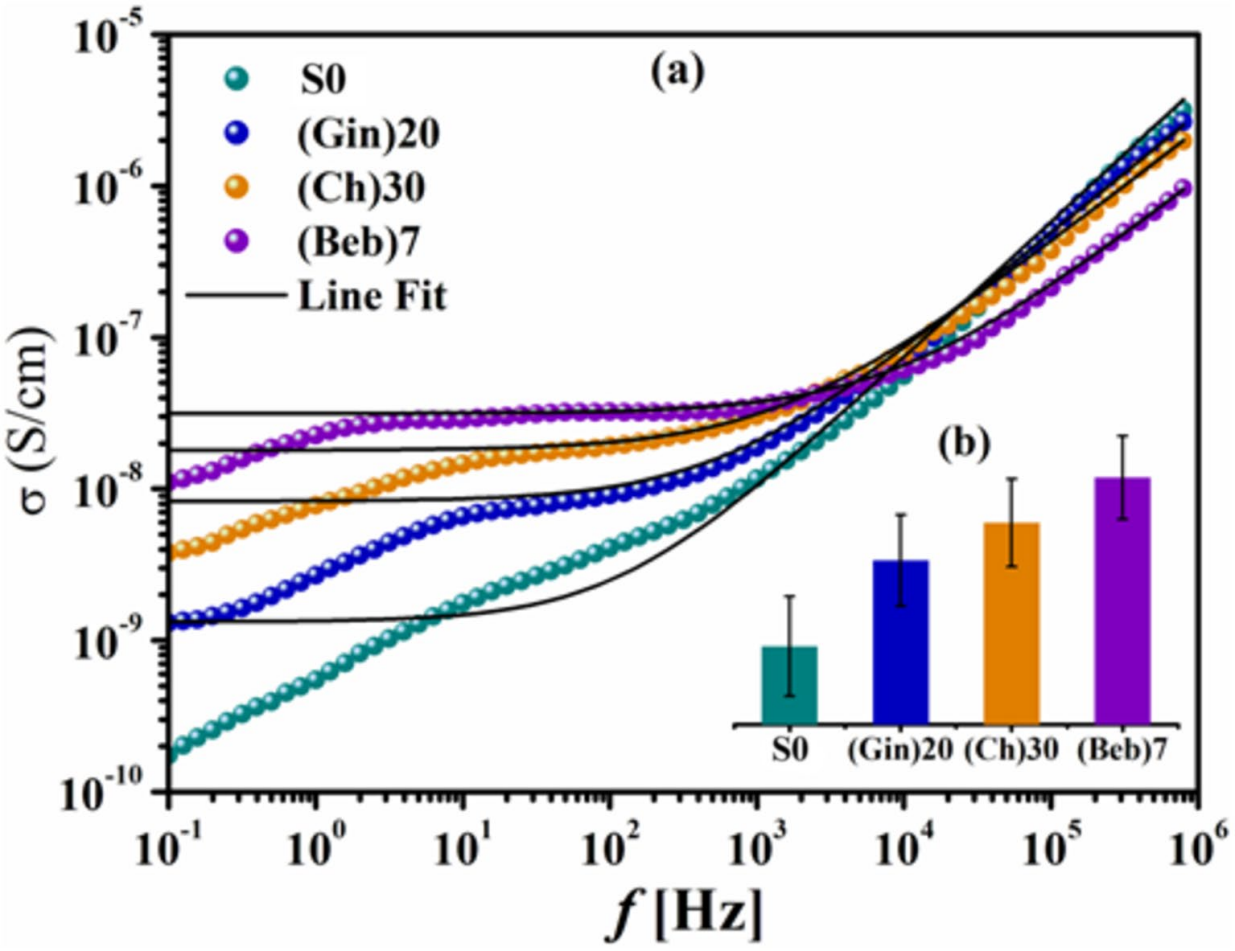
Frequency-dependent conductivity (σ ) of SBR-based composites at 30 °C, and the fit lines in accordance with the power law.

Figure 8a illustrates the variation of electrical conductivity (σ) in SBR-based vulcanizates as a function of frequency. All samples exhibit a power-law increase in conductivity with frequency, which is associated with hopping conduction and interfacial polarization in elastomeric matrices^57^. (Beb) 7 exhibits the highest conductivity within the spectrum due to the conjugated ionic structure of berberine, which facilitates the movement of charge carriers and promotes space charge accumulation^60^. (Gin) 20, and (Ch) 30 exhibit intermediate conductivity values, indicating the influence of polar phytochemicals in ginger and proton-conducting pathways in chitosan on these measurements^56,58^. The blank sample (N0) exhibits the lowest conductivity, indicating that bioactive material facilitates electrical transport.(Beb)7 exhibits the largest dielectric loss ε″, indicating rapid movement of charge carriers and significant friction at the contact^61^., (Gin) 20, and, (Ch) 30 follow with moderate declines, although N0 experiences minimal dissipation. The results indicate that dielectric properties vary with frequency and that berberine serves effectively as a functional material used for applications requiring enhanced permittivity and regulated energy dissipation.

Besides, the insert Fig. 7b illustrates the DC conductivity (σ_dc) determined in the low-frequency range, the plateau region through power law fitting. At room temperature (30 °C), these values rose from 1.55 × 10^− 9^ S/cm for SBR vulcanizates without bioactive agent (N0) to 3.35 × 10^− 8^ S/cm for composite (Beb)7. The composites (Gin)20, and (Ch)30 have moderate values (8.82 × 10^− 9^ S/cm are 1.92 × 10^− 8^ S/cm).However, the outcomes of these composites clearly support the usage of these bioactive agent-based SBR composites as antistatic materials^62^. On the other hand, as discussed earlier, (Beb)7 composite exhibits the highest σ_dc, indicating its potential utility in shielding against electromagnetic interference (EMI) and static electricity^62–65^.

**Fig. 9:**
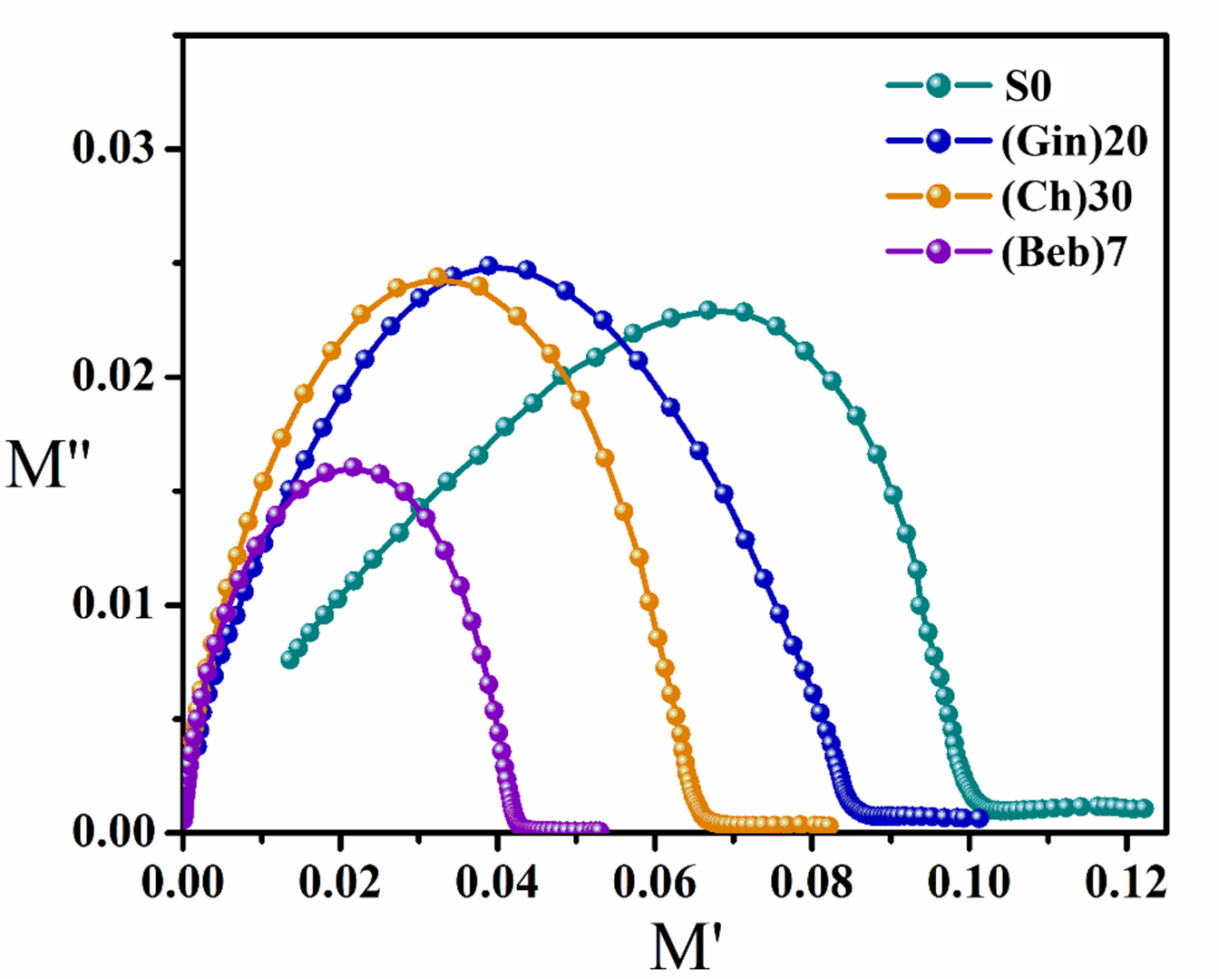
Cole-Cole plot of M″ vs. M′ for SBR composites.

Figure 9 presents the intricate electric modulus spectra (M″ vs. M′) for SBR-based composites. This provides insight into the mechanism of their dielectric relaxation. All samples exhibit distinct semicircular arcs, indicating non-Debye relaxation behavior and a distribution of relaxation times typical of elastomeric systems^57^. The (Beb)7 sample exhibits the lowest arc signifying considerable interfacial polarization due to berberine’s ionic structure and conjugated domains, along with high conductivity^54^.

(Gin)20, and (Ch)30 exhibit intermediate arc profiles, indicating the influence of polar phytochemicals and hydrogen-bonding interactions on segmental mobility and charge carrier dynamics^58,59^. The N0 sample (blank sample) exhibits extensive arc due to its non-polar nature and minimal dielectric activity. The variation in peak positions and arc curvature among the samples indicates that bioactive agents significantly alter the relaxation by introducing localized dipolar sites, ionic channels, and interfacial heterogeneity. The findings align with previous studies on polymer vulcanizates that employed electric modulus analysis to distinguish bulk conductivity from interfacial phenomena^65^.

### Bioassay tests for the antimicrobial rubber products

#### Cytotoxic effect

**Fig. 10:**
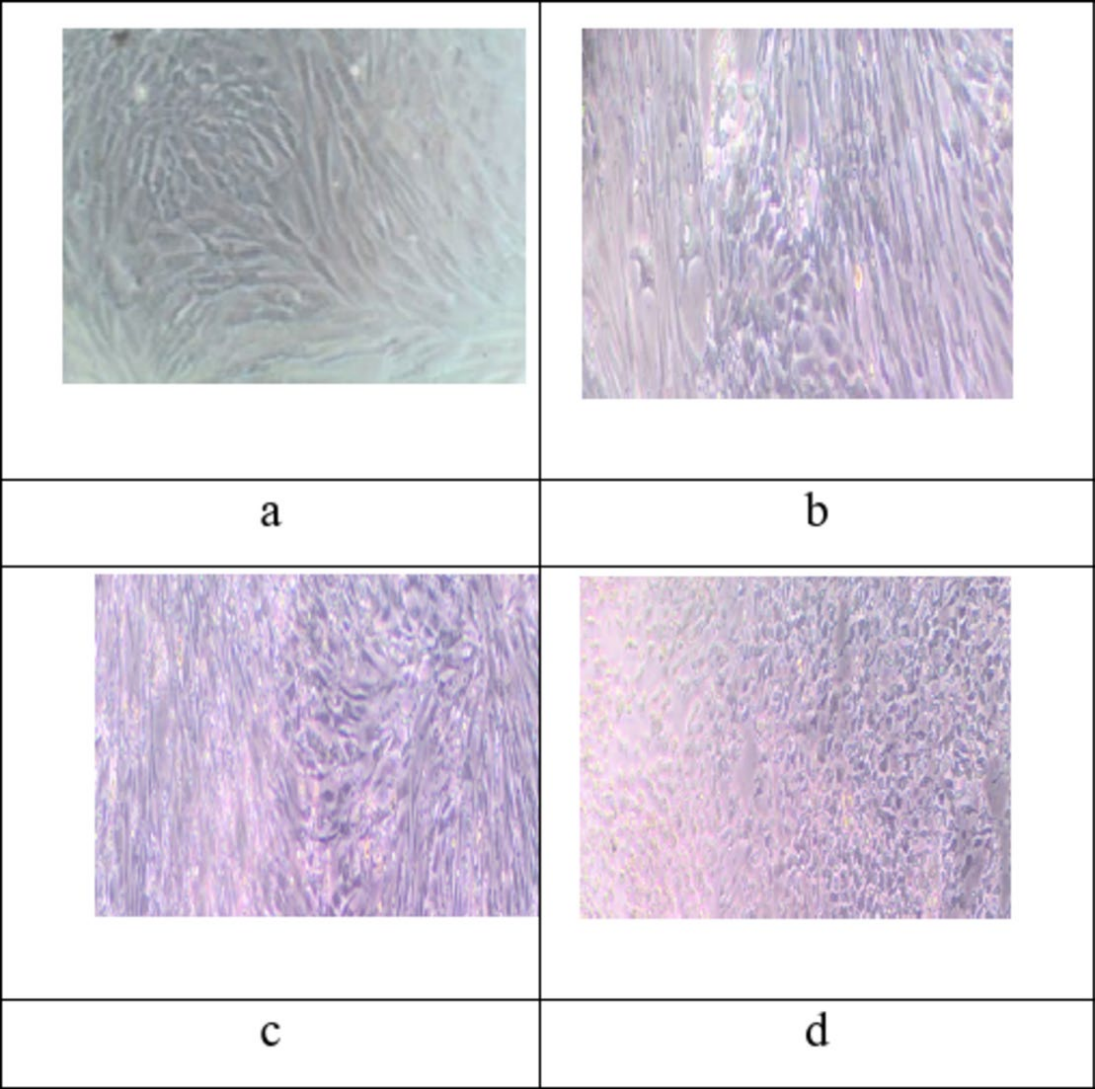
Cytotoxic activity of different SBR vulcanizates : a- control b- loaded with Chitosan c- loaded with Berberine d- loaded with Ginger.

The cytotoxic effect of the investigated rubber vulcanizates loaded with different antimicrobial agents was tested towards the normal human skin cell line: **BJ1 Fibroblast** and illustrated in Table 4; Fig. 10.

**Table 4:** Cytotoxic activity of different SBR Rubber vulcanizates towards human normal BJ1 Fibroblast cells.

Sample Code	Remarks
SBR/Chitosan	N.A.
SBR/Ginger	N.A.
SBR/Berberine	N.A.
DMSO	N.A.
Negative control	N.A.

The data in Table 4; Fig. 10 indicate that the investigated SBR rubber vulcanizates loaded with different antimicrobial agents are safe and have nil cytotoxicity. Moreover, there is no effect of the strong color and odors of Ginger extract on the normal human fibroblast cell line (BJ1) under investigation.

The cytotoxicity of the prepared vulcanizates towards human normal BJ1 Fibroblast cells indicated good safety of these rubber products. This result indicates that the prepared SBR vulcanizates did not harm human normal BJ1 Fibroblast cells in a lab test, which is a positive sign for the biocompatibility and safety of these particular rubber products for certain applications.

The antimicrobial testing lacks Without a comprehensive description of the methodology and a scientifically sound display of the data, the findings cannot be properly validated.

#### Antimicrobial activity

**Fig. 11:**
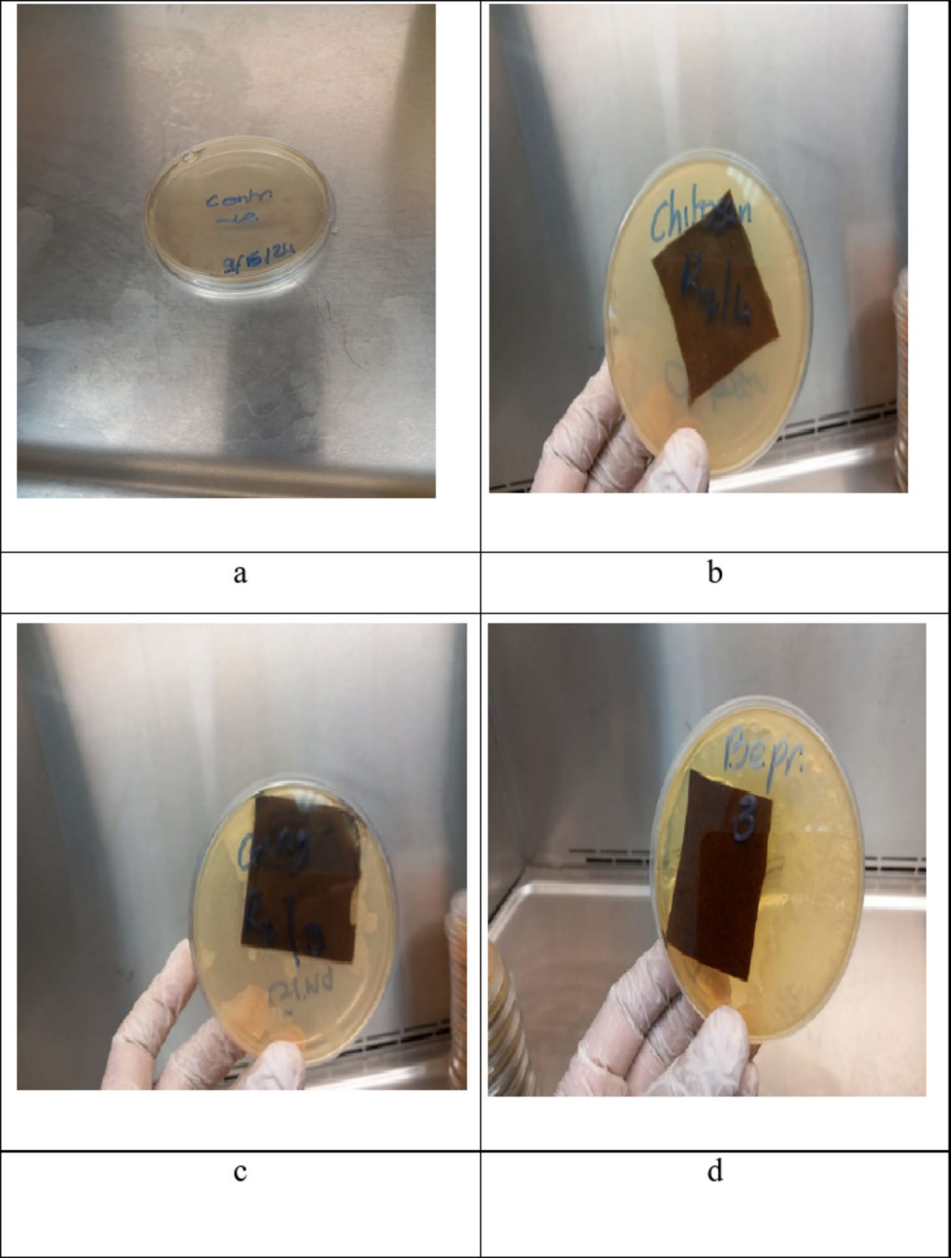
Antimicrobial activities of different SBR vulcanizates towards *Staphylococcus aureus* Gram-positive bacteria: a- control b- loaded with Chitosan c- loaded with Berberine d- loaded with Ginger.

1-Antimicrobial Activity Against Staphylococcus aureus (Gram-positive):

Figure 11 illustrates the antimicrobial activity of SBR rubber vulcanizates loaded with different antimicrobial agents against *Staphylococcus aureus* (Gram-positive), compared to a control (unloaded SBR rubber). Among the tested formulations, SBR rubber loaded with chitosan exhibited the highest growth inhibition potency. SBR rubber loaded with berberine showed moderate activity, while the ginger-loaded SBR rubber demonstrated the lowest inhibitory effect against *S. aureus*.

2-Antimicrobial Activity Against *Escherichia coli* (Gram-negative)

**Fig. 12:**
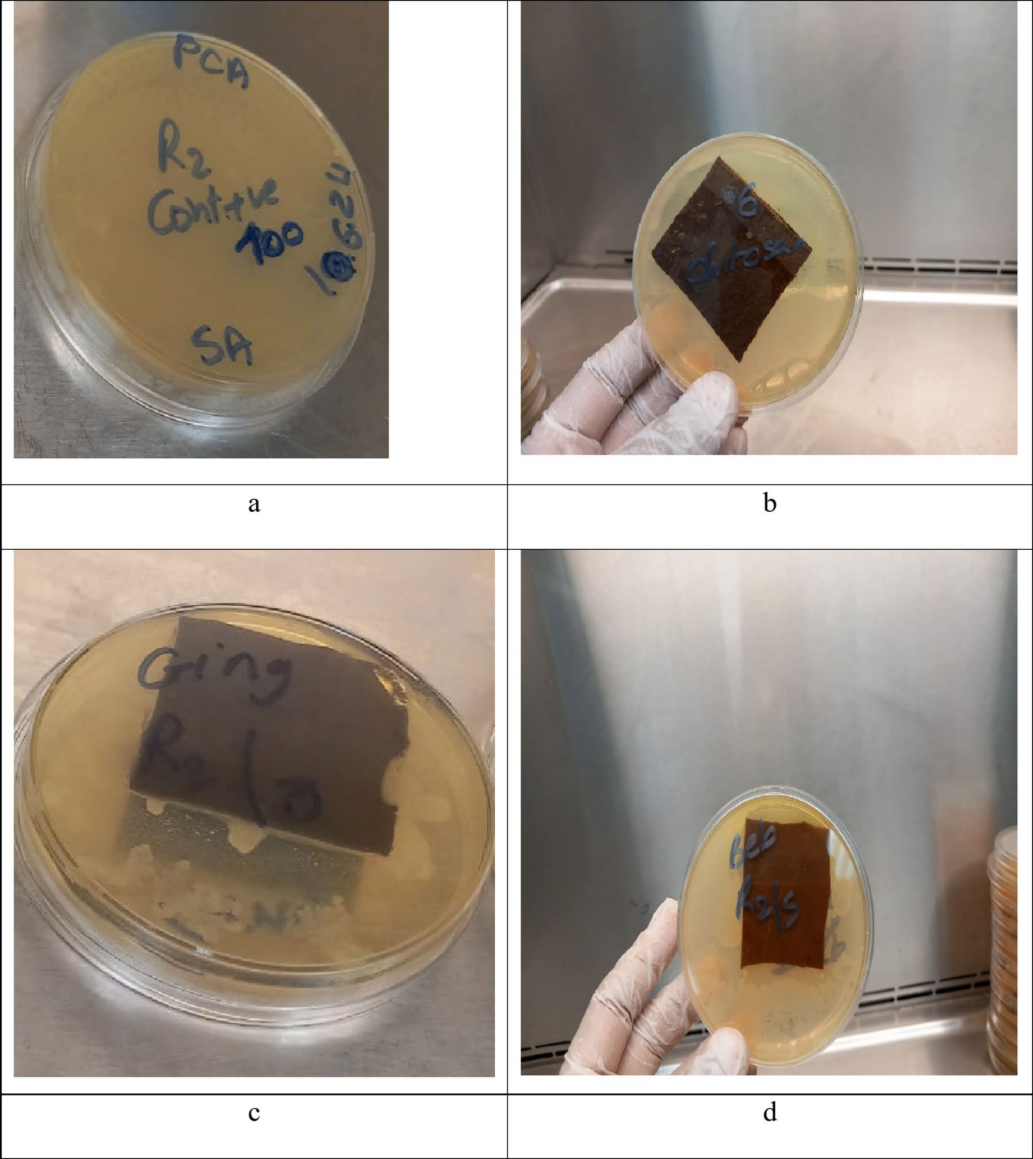
Antimicrobial activities of different SBR vulcanizates towards *Escherichia coli* Gram-negative bacteria: a- control, b- loaded with Chitosan, c- loaded with Berberine d- loaded with Ginger.

Figure 12 presents the antimicrobial activity of the same SBR rubber vulcanizate formulations against *Escherichia coli* (Gram-negative). In contrast to the results observed for *S. aureus*, berberine-loaded SBR rubber was the most potent against *E. coli*, followed by chitosan-loaded SBR rubber. Ginger-loaded SBR rubber again showed the lowest efficacy in inhibiting bacterial growth.

So, the antibacterial efficiency against *S. aureus* Gram +ve and *E. coli* Gram -ve was successfully improved by the loading of the tested antimicrobial agents into the SBR rubber vulcanizates.

The objective of this research was to enhance the antimicrobial properties of SBR rubber vulcanizates by loading chitosan, berberine and ginger extract as antimicrobial agents. These results are in accordance with many previous works^6,10,11^, which reported that the incorporation of different antimicrobial agents significantly improved both the antibacterial performance against different types of bacteria, and demonstrating the potential of these agents as functional bio-fillers in sustainable rubber composites.

The main aim of this research was to enhance the antimicrobial properties of SBR rubber vulcanizates by loading chitosan, berberine and ginger extract as antimicrobial agents. These results are in accordance with previous works^64^, which stated that the incorporation of chitosan nanoparticles significantly improved both the antibacterial performance against *E. coli* and *S. aureus*, and demonstrating the potential of chitosan nanoparticles as functional bio-fillers in sustainable rubber composites. A greener approach for producing antimicrobial rubber vulcanizates has gained significant importance and demand for personal care due to their less toxic effects on health and the environment In comparison to synthetic drugs, the naturally existing materials including extracts and essential oils of plants have significant applications for antimicrobial rubber. Additionally, a number of animal extracts are also used as antimicrobial agents include chitosan, alginate, collagen hydrolysate to prepare naturally treated antimicrobial rubber. This work focuses on the comparative performance of antimicrobial agents between synthetic and natural materials. Rubber with synthetic substances cause health and environmental concerns whereas rubber treated with natural compositions are more safe and eco-friendly. Finally, it is concluded that rubber vulcanizates with natural antimicrobial compositions may be a better alternative and option as functional rubber. So, the vulcanization of SBR rubber formulations occurred at the expected optimal cure time. Therefore, the antibacterial characteristics of the compounded SBR are improved by the use of plant extracts and pharmaceutical medications, which have no adverse effects.

As a consequence, a novel rubber composite based on styrene-butadiene rubber (SBR) was formulated by the incorporation of chitosan, berberine, and ginger as multifunctional natural additives. Chitosan serves to enhance interfacial adhesion while also conferring antimicrobial properties, whereas berberine imparts supplementary bioactive reinforcement and thermal stability. The inclusion of ginger extract bestows antioxidant characteristics and enhances the overall durability of the composite material. Collectively, these environmentally benign constituents yield a distinctive and sustainable SBR rubber composite that exhibits enhanced mechanical, anti-aging, and functional performance. So, the ecofriendly investigated compounds have good advantages for incorporating in SBR to obtain safety rubber products for their properties, based on the obtained results, the interactions between the added bioactive components (ginger extract, chitosan, and berberine) and the SBR elastomer matrix can be explained as follows:

- Chitosan (30 phr):
- Chitosan has amino and hydroxyl groups that can form weak electrostatic bonds with the SBR chains. These bonds help the materials work better together and create more physical links between them. This explains why the material becomes stronger, swells less, and responds better to electric fields. The chitosan also has a polar structure, which allows for easier movement of protons and leads to a slight increase in how well it conducts electricity and how it reacts to electric charges.
- Ginger extract (20 phr):

Ginger has compounds like phenolics, flavonoids, and ketones. These are types of plant chemicals that are polar. They can help improve how well materials stick together, which makes the material stronger and more heat-resistant. Because they are bioactive, they also help make the rubber-like materials more effective at fighting bacteria.

- Berberine (7 phr):

Berberine has a structure that is both ionic and aromatic, which helps create strong polarization at the interfaces inside the rubber-like material. This explains the big improvement in how the material handles electric charges, its ability to store electrical energy, and how well it conducts electricity. Its structure also helps electrons and ions move more easily, making it the best at preventing static electricity among all the materials tested.

## Conclusion

Styrene butadiene rubber (SBR) incorporating plant extracts from ginger, as well as the conventional drug berberine and chitosan, is highly promising for the production of safe, green antimicrobial rubber products. They were cured at expected optimal cure time. Therefore, the antimicrobial characteristics improved.

The investigated SBR vulcanizates demonstrated excellent mechanical properties both before and after thermal oxidative aging, and they had no cytotoxic effects on human fibroblast cells. The investigated vulcanizates showed significant inhibitory effects against a range of bacteria and fungi. It is advised to create and manufacture a kind of personal protection product, along with other safety rubber items, using SBR vulcanizates that contain chitosan, ginger, or berberine.

Also, the incorporation of bioactive agents into the SBR matrix significantly alters its electrical and dielectric properties. Berberine at 7phr exhibited superior performance across all measured parameters. This resulted from the conjugated ionic structure of berberine and its considerable interfacial polarization. Chitosan at 30phr and Ginger at 20phr exhibited moderate enhancements attributable to the influence of polar phytochemicals and functional groups that facilitate proton transfer. The investigation of the electric modulus further confirmed the presence of non-Debye relaxation behavior and distributed relaxation. SBR composites based on berberine, chitosan, and ginger are an excellent solution for antistatic applications and flexible electronics, where enhanced permittivity, conductivity, and controlled energy dissipation are crucial. Furthermore, developed rubber vulcanizates showed good antimicrobial efficacy towards the test bacteria and fungi strains. Results showed that the loaded antimicrobial agents significantly improve the antibacterial performance of the prepared SBR rubber vulcanizates while maintaining biodegradability, thus offering a novel, sustainable approach to fabricating functional rubber foams for healthcare and environmental applications.It is recommended that, the investigated eco-friendly ingredients create a unique, sustainable SBR vulcanizates with improved mechanical, antimicrobial, electrical, and functional performance. 

## Data Availability

The data that support the findings of this study are available from the corresponding author upon reasonable request.
